# Biological and epidemiological evidence of anti-allergic effects of traditional Japanese food *ume* (*Prunus mume*)

**DOI:** 10.1038/s41598-018-30086-5

**Published:** 2018-08-03

**Authors:** Ryohei Kono, Misa Nakamura, Sachiko Nomura, Naomi Kitano, Tomoko Kagiya, Yoshiharu Okuno, Ken-ichi Inada, Akihiko Tokuda, Hirotoshi Utsunomiya, Masami Ueno

**Affiliations:** 1 Department of Strategic Surveillance for Functional Food and Comprehensive Traditional Medicine, Wakayama Medical University, 811-1 Kimiidera, Wakayama City, Wakayama 641-0012 Japan; 2 Department of Rehabilitation, Osaka Kawasaki Rehabilitation University, 158 Mizuma, Kaizuka City, Osaka 597-0104 Japan; 3 Research Center for Community Medicine, Wakayama Medical University, 811-1 Kimiidera, Wakayama City, Wakayama 641-0012 Japan; 4 Department of Public Health, School of Medicine, Wakayama Medical University, 811-1 Kimiidera, Wakayama City, Wakayama 641-0012 Japan; 5 Faculty of Health Science, Kansai University of Health Science, 2-11-1 Wakaba, Kumatori-cho, Sennan-gun, Osaka 590-0482 Japan; 6 Department of Applied Chemistry and Biochemistry, National Institute of Technology, Wakayama Collage, 77 Noshima, Nada, Gobo, Wakayama 644-0023 Japan; 7 Department of Pathology, Fujita Health University School of Medicine, 1-98 Dengakugakubo, Kutsukake-cho, Toyoake, Aichi 470-1192 Japan

## Abstract

Japanese apricot (*Prunus mume*; *ume*) is a traditional food in Japan that has been shown to have various beneficial health effects. There is some evidence to suggest that *ume* is also effective against allergic disease. Here, we conducted a cross-sectional epidemiological pilot study to examine the association between *ume* intake frequency and allergic symptoms including rhinitis in 563 adults (288 men and 275 women) who resided in Wakayama, Japan. After adjusting for age, present illness and medication, women with high *ume* intake had significantly lower odds ratio (OR) for the presence of symptoms of allergy [OR: 0.49 with 95% confidence interval (CI): 0.25–0.97]. Therefore, we investigated the anti-allergic effect of *ume* on passive cutaneous anaphylaxis (PCA) reaction in immunoglobulin E (IgE)-sensitized mice. The animal study demonstrated that oral administration of *ume* extract attenuated the PCA reaction and mast cell degranulation. Furthermore, RBL-2H3 mast cells were used to identify anti-allergic *ume* compounds. The following *ume* compounds inhibited IgE-mediated mast cell degranulation: vanillin, syringic acid, protocatechuic aldehyde, lyoniresinol and *p*-coumaric acid. These results suggested that *ume* has the potential to inhibit mast cell degranulation and may be associated with reduced risk of allergic symptoms in women.

## Introduction

The number of people suffering from an immunoglobulin E (IgE)-mediated (type I) response to an allergen has increased worldwide. Allergic reactions including hay fever, food allergy and bronchial asthma occur due to environmental antigens (known as allergens) such as pollen^1^, certain foods^2^ and house dust mites^3^. Because allergy symptoms can result not only in a decline in quality of life but also in life-threatening reactions, allergies have become a social problem. Development of Japanese cedar or Japanese cypress pollen allergy (pollinosis) has recently increased in Japan. The most common cause of pollinosis in Japan is Japanese cedar. A nationwide survey found that the prevalence of Japanese cedar pollinosis increased from 16.2% in 1998 to 26.5% in 2008^4^. Functional foods, defined as foods that can provide additional health benefits beyond that of traditional nutrients they contain, have attracted attention as a potential solution, and some studies have focused on elucidating anti-allergic functions of food components. For example, catechin derived from Japanese green tea^5,6^ or flavonoid derived from citrus fruits were demonstrated to have potential anti-allergic effects^7–9^. Identification and adoption of anti-allergic foods may be one strategy to decrease the severity of some allergic symptoms, such as those associated with pollinosis.

In research on the anti-allergic effect of functional foods, mast cells are frequently used to detect an active compound from foods or to clarify the mechanism of anti-allergic effects *in vitro*. Mast cells play an important role in immediate allergic reactions^10^. Antigen-specific IgE antibody binds to high-affinity IgE receptor (FcεRI) on the mast cell membrane^11^. In IgE-dependent mast cell activation, antigen-mediated cross-linking of cell surface FcεRI induces degranulation and the release of allergic chemical mediators such as histamine, cytokines and proteases^12^. Histamine not only regulates several essential events in the allergic response but also plays an important role both physiologically and pathologically^13^. Because β-hexosaminidase is also released along with histamine upon mast cell degranulation, this enzyme is typically used as a marker for mast cell degranulation in *in vitro* studies. Therefore, anti-allergic compounds are identified using inhibition of mast cell degranulation as an indicator. In *in vivo* studies, inhibitory testing of natural compounds or food extracts is usually performed by the passive cutaneous anaphylaxis (PCA) test as an animal model of IgE-mediated allergic response^14–16^. Anaphylaxis is triggered in response to allergen exposure following IgE sensitization^17^. When mast cells are exposed to an antigen, IgE binding brings FcεRI receptors on mast cells in close proximity, allowing cross-linking between receptors. Receptor cross-linking then triggers the release of chemical mediators from mast cells and basophils^18^. Therefore, the PCA test is widely used as *in vivo* model of type I allergy induced by the release of chemical mediators.

*Prunus mume* is considered a traditional food and medicine in Asian countries such as Japan and China. In Japan, *P*. *mume*, called ‘*ume*’ in Japanese, is consumed dried or pickled with salt and in *ume*-containing liquor or soft drinks. Recently, studies have suggested that *ume* has the potential to prevent osteoporosis^19,20^, atherosclerosis^21^ and *Helicobacter pylori* infection^22^. *Ume* seed extract is known to have various functions including a protective effect in human ovarian granulosa cells against oxidative stress^23^ and inhibition of adult T cell leukaemia proliferation^24^. Research on the medicinal properties of *ume* extract is as important as those of processed *ume* because *ume* seed components are transferred into pulp, liquor or soft drinks during processing. Here, to understand the effect of *ume* intake, we conducted a pilot study targeting apparently healthy community-dwelling people to investigate the association between frequency of *ume* intake and allergic symptoms in a specific area in Japan. Then, to clarify the mechanism, the effect of *ume* seed extract was studied by the PCA test in mice. Bioactive *ume* compounds were detected by guided isolation based on the inhibitory effect of *ume* seed extract on allergen-mediated β-hexosaminidase release from mast cells and the mechanisms of compounds were discussed.

## Results

### Association between frequency of ume intake and allergic symptoms

Table 1 shows the distribution of *ume* intake and description of allergy symptoms. The median of age was 51 years (mean ± SD 50.8 ± 13.5) in men and 63 years (mean ± SD 60.7 ± 12.4) in women. The proportions of men and women with allergic symptoms were 21.2% (61/288) and 26.9% (74/275), respectively. Among allergic symptoms of men and women, pollinosis accounted for 70.5% (43/61) and 82.4% (61/74), respectively. A linear tendency was observed in the proportion of women with allergic symptoms among the 3 categories of *ume* intake: the higher the frequency of *ume* intake, the lower the proportion of women with symptoms of allergy (Table 1). After adjusting for age, present illness excluded current allergic disease and medication, women with high *ume* intake had significantly lower OR for the presence of allergy symptoms (OR 0.49, 95% CI 0.25, 0.97) (Table 1).

**Table 1:** Association between frequency of *ume* intake and allergy symptoms.

	Frequency of *ume* intake	Allergy symptoms	Crude				Multivariate*			
		Yes (%)/No (%)	ORs	95% CIs		*P-*value	ORs	95% CIs		*P-*value
				Lower	Upper			Lower	Upper	
Men (n = 288)	High	20 (22.0)/71 (78.0)	0.94	0.49	1.79	0.848	1.45	0.71	2.99	0.312
	Middle	11 (16.4)/56 (83.6)	0.66	0.31	1.41	0.278	0.88	0.39	1.97	0.751
	Low	30 (23.1)/100 (76.9)	1.00				1.00			
Women (n = 275)	High	16 (17.4)/76 (82.6)	0.40	0.21	0.78	0.007	0.49	0.25	0.97	0.040
	Middle	18 (26.9)/49 (73.1)	0.70	0.36	1.35	0.287	0.76	0.38	1.53	0.446
	Low	40 (34.5)/76 (65.5)	1.00				1.00			

*Adjusted for age (5 categories), presence/absence of illness (except for allergic disease) and presence/absence of medication (including anti-allergic drugs).OR: odds ratio, CI: confidence interval.

High: ≥1 *ume* daily, Middle: <1 *ume* daily, Low: ≤2 *ume* weekly or none.

### *In vivo* effects of ume extract on PCA reaction

**Figure 1:**
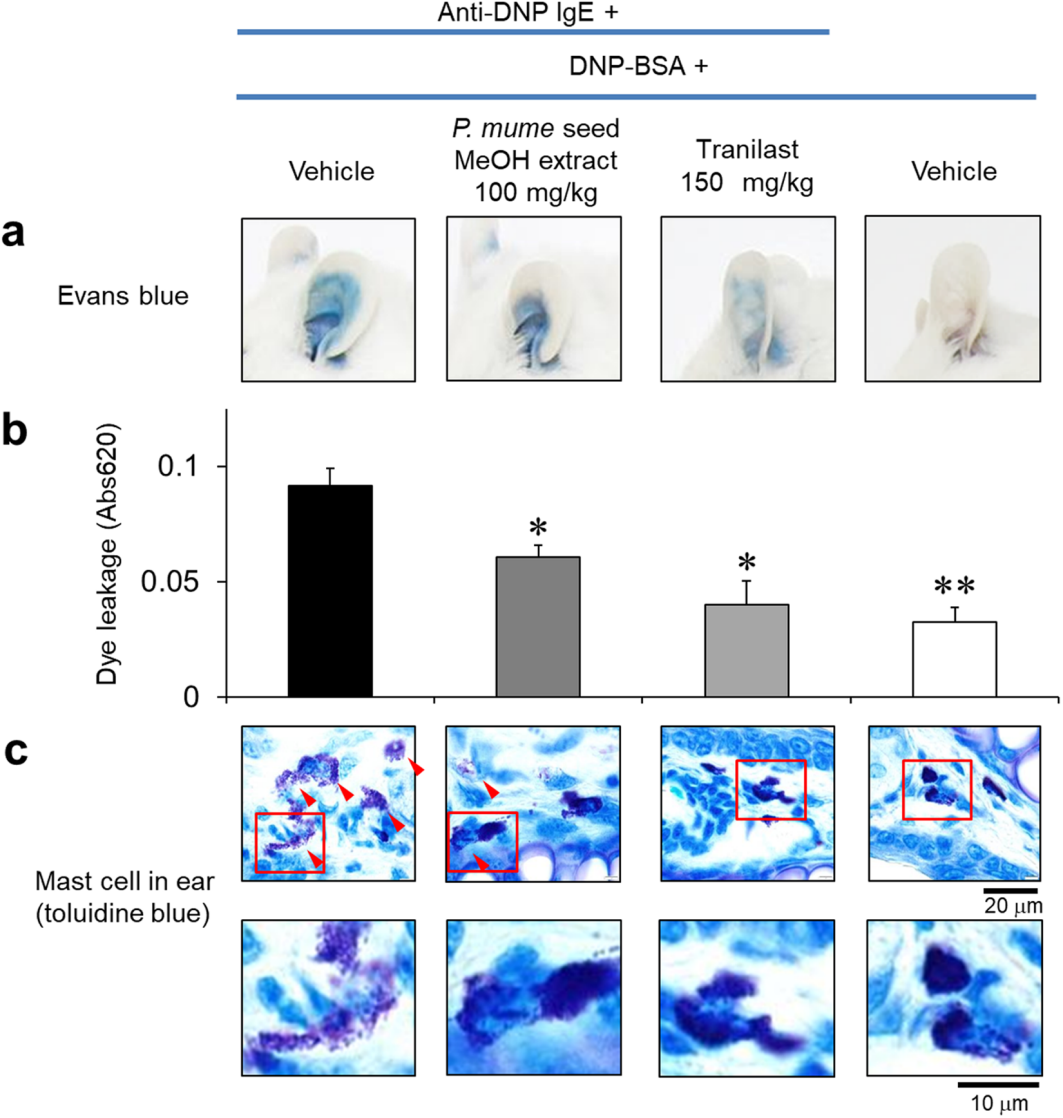
Inhibitory effect of *ume* seed extract on PCA reaction in mice. (**a**) Images of extravasated Evans blue dye from mouse ears after PCA reaction. TL was used as an anti-allergic positive control. (**b**) Amount of extravasated Evans blue dye from mouse ears. Data represent the mean ± SE (n = 5–7) of the average absorbance calculated from left and right mouse ears. *p < 0.05, **p < 0.001, compared with vehicle. (**c**) Ear tissue sections were stained with toluidine blue to visualize mast cell degranulation. Lower images are a magnified view of the area enclosed by the red frame in upper images (magnification, 400×). Scale bar: 20 µm (upper images) or 10 µm (lower images). Red arrows indicate degranulated cells.

We next determined whether *ume* attenuates IgE-mediated PCA reactions in the mouse ear. A methanol extract of *ume* seed, which included the basic ingredient of *ume* contained in processed foods, was used in this study. When mouse ears were sensitized with dinitrophenyl (DNP)-specific IgE and intravenously challenged with the antigen 2,4-dinitrophenylated bovine serum albumin (DNP-BSA), PCA reaction was concomitantly induced with rapid capillary dilatation and increased vascular permeability in mouse ears. The PCA reaction was visualized by leakage of Evans blue dye into the reaction site of ears. However, in mice treated with oral administration of methanol extract of *ume* seed, vascular permeability of the ears was attenuated, as judged from the extent of blue staining in the ear (Fig. 1a) and intensity of Evans blue dye extracted from the ears (*p* < 0.05, Fig. 1b).

We further histologically examined mast cells in the mouse ear after antigen challenge. Ear tissue sections from IgE-sensitized mice challenged with DNP-BSA were stained with toluidine blue in order to observe mast cells by light microscopy. In the absence of IgE sensitization (negative control), ear tissue contained intact toluidine blue-positive mast cells (Fig. 1c). In the presence of IgE sensitization (positive control), degranulated mast cells were identified in histological tissue sections after 1-h DNP-BSA challenge. These degranulated mast cells decreased with oral administration of tranilast (TL, anti-allergic drug) and *ume* seed methanol extract before antigen challenge.

### Inhibitory effect of ume compounds on mast cell degranulation and its mechanisms

**Figure 2:**
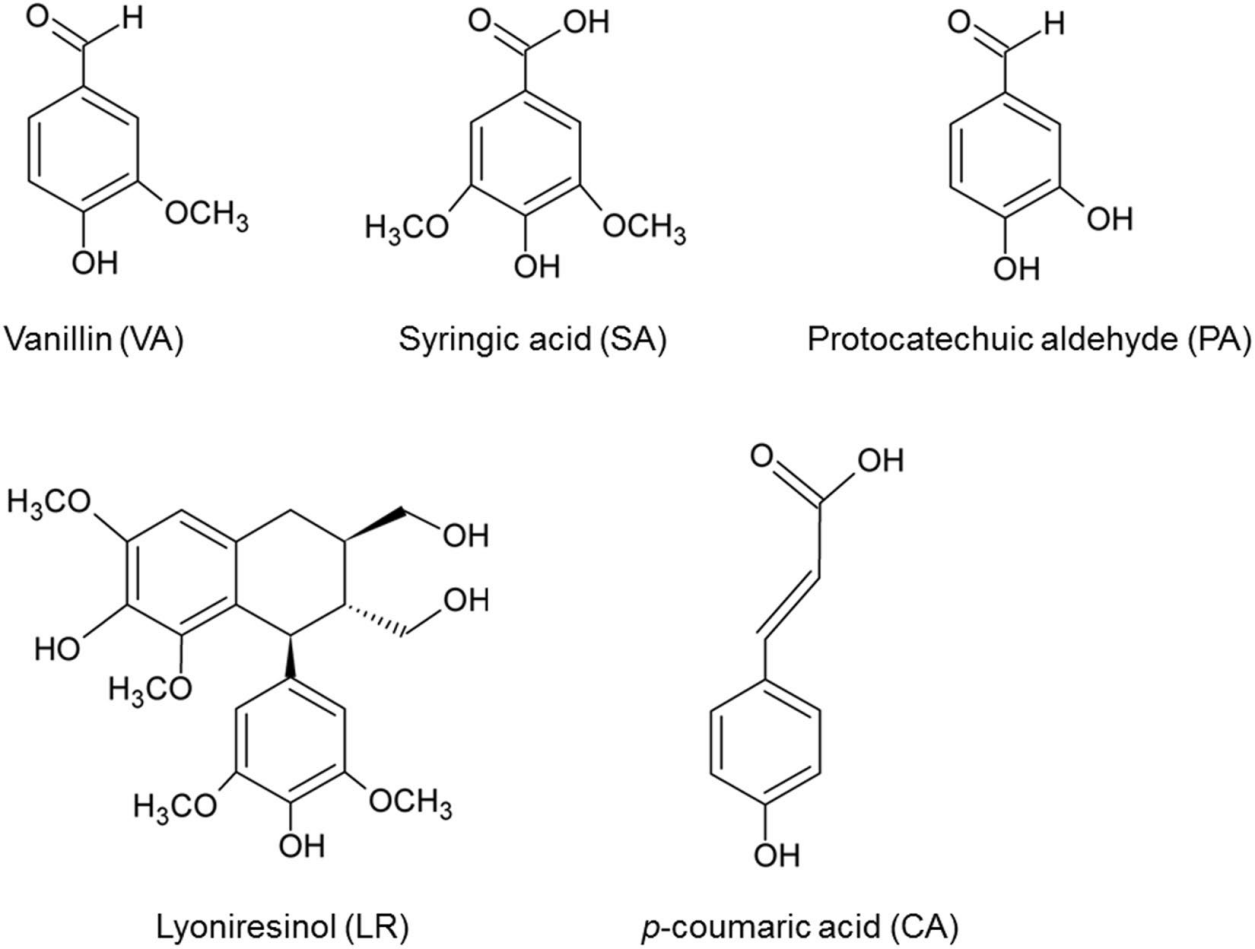
Determination of the chemical structures of active compounds isolated from *ume* seed. Active compounds were isolated from *ume* seed extract based on their inhibitory effect on antigen-induced β-hexosaminidase release. Isolated compounds were identified as vanillin (VA), syringic acid (SA), protocatechuic aldehyde (PA), lyoniresinol (LR) and *p*-coumaric acid (CA).

**Figure 3:**
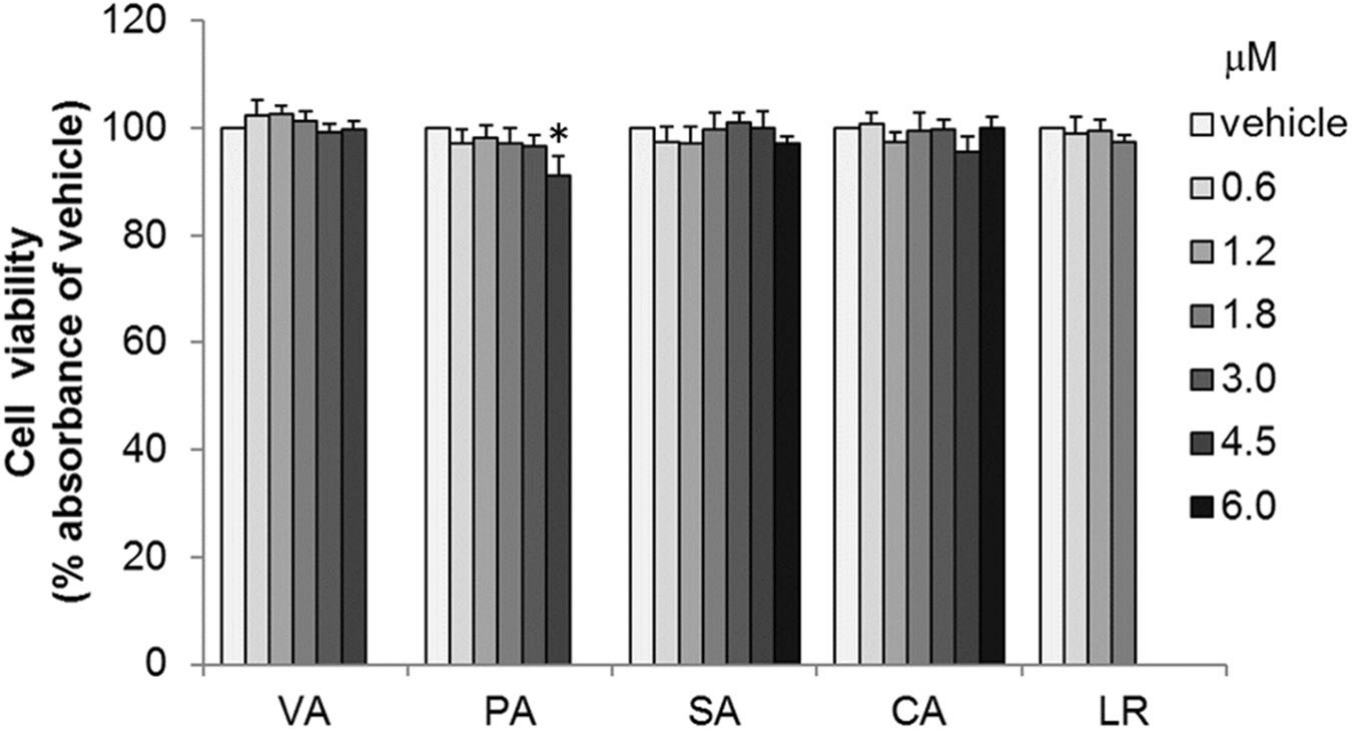
Effect of *ume* compounds on viability of RBL-2H3 cells. RBL-2H3 cells were treated with SA and CA at concentrations up to 6 mM, VA and PA at concentrations up to 4.5 mM and LR at concentrations up to 1.8 mM. Cell viability is expressed as a percentage relative to the vehicle. Data are expressed as the mean ± SD of triplicate wells (n = 3). *p < 0.05, compared with vehicle.

To determine the active compounds from methanol extract of *ume* seed, methanol extracts were further separated into hexane, dichloromethane, ethyl acetate and water fractions. These extracts were used to screen anti-allergic fractions guided by an inhibitory effect on β-hexosaminidase release of RBL-2H3 mast cells induced by antigen-antibody reaction. Because dichloromethane and ethyl acetate fractions exhibited an inhibitory effect on β-hexosaminidase release, these fractions were further separated and purified by column chromatography. Finally, we identified the following five active compounds from *ume* seeds by high-resolution electrospray ionisation mass spectrometry (HR-ESI-MS) and proton nuclear magnetic resonance (^1^H-NMR): vanillin (VA), syringic acid (SA), protocatechuic aldehyde (PA), lyoniresinol (LR) and *p*-coumaric acid (CA) (Fig. 2). To further examine whether these active compounds were contained in pickled *ume*, quantitative analysis was performed by high-performance liquid chromatography (HPLC) and all compounds were contained in pickled *ume*. No cytotoxicity was observed in the sample concentration range adopted in this study (Fig. 3). Since PA was found to affect cell viability at high concentration (4.5 mM), PA was applied at concentrations lower than 4.5 mM in subsequent studies.

**Figure 4:**
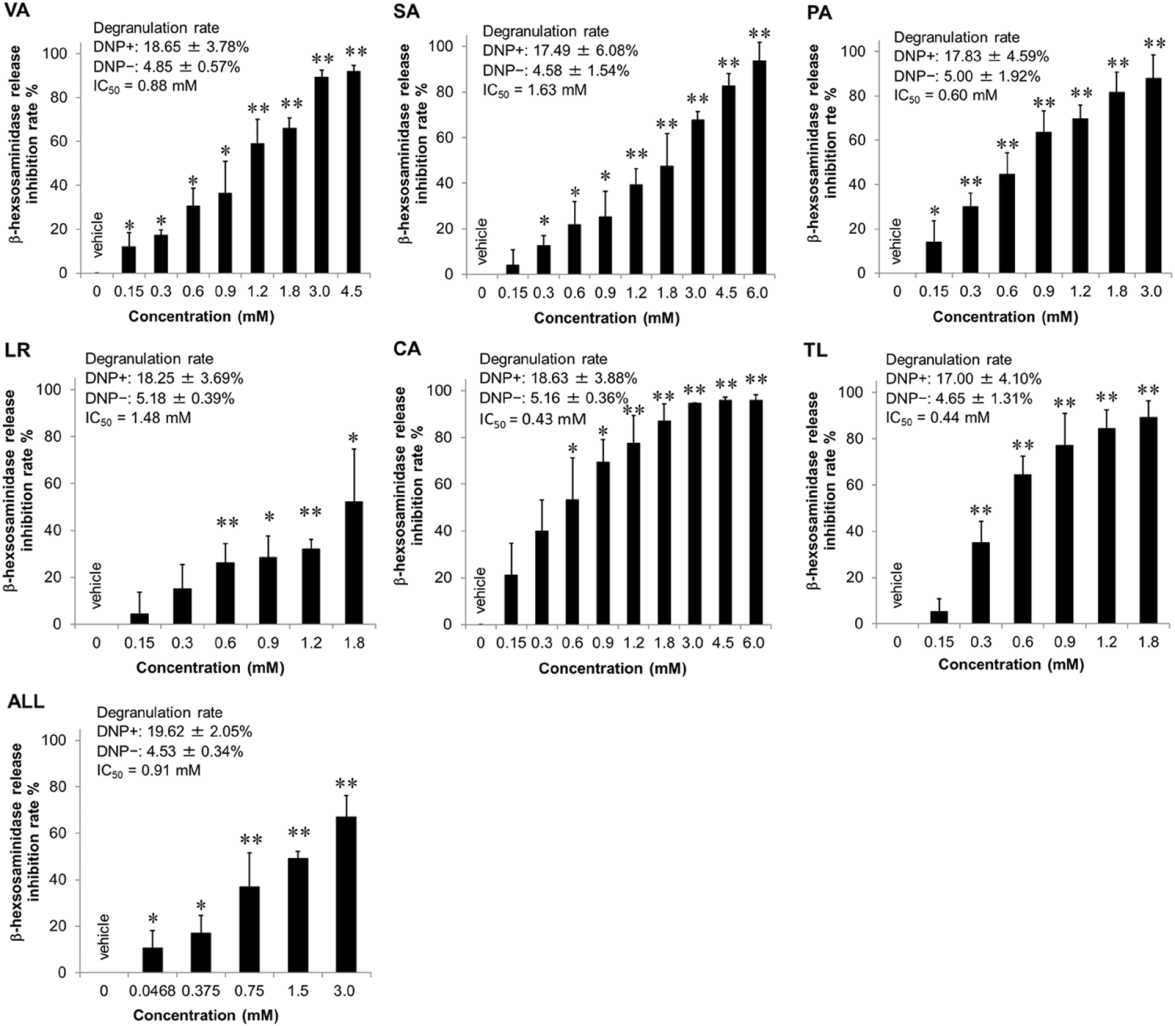
Inhibitory effects of active compounds derived from *ume* on antigen-induced degranulation of RBL-2H3 cells. IgE-sensitized RBL-2H3 cells were stimulated with DNP-BSA in the presence of *ume* seed extracts or vehicle (0.1% DMSO). TL was used as an anti-allergic positive control. Data represent the mean ± SD (n = 3) of β-hexosaminidase release inhibition rate of three independent experiments. ALL: equal molar concentrations of VA, SA, PA, LR and CA; ALL concentration: the total concentration of the five compounds; IC_50_: 50% inhibitory concentration on β-hexosaminidase release. Degranulation rates of maximum response when cells are treated with vehicle and DNP-BSA (DNP+) or minimum response when cells are treated with vehicle alone (DNP-) and IC_50_ are shown in the figures. *p < 0.05, **p < 0.001, compared with vehicle.

**Figure 5:**
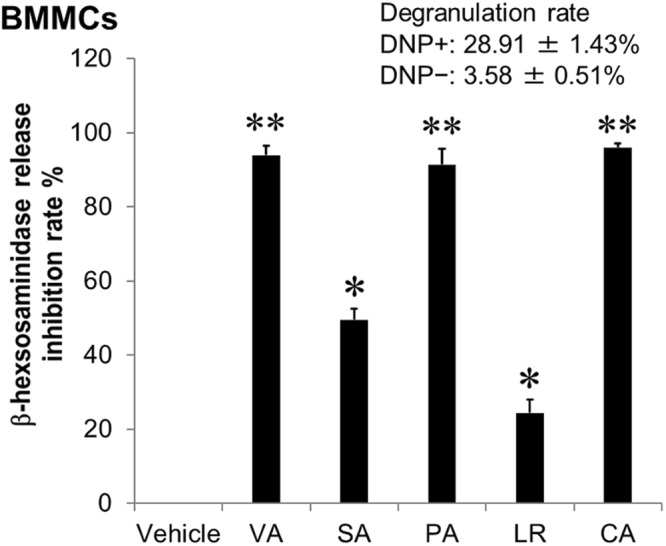
Inhibitory effects of active compounds derived from *ume* on antigen-induced degranulation of BMMCs. IgE-sensitized BMMCs were stimulated with DNP-BSA in the presence of active compounds derived from *ume* seed at 600 μM or vehicle (0.1% DMSO). Data represent the mean ± SD (n = 3) of β-hexosaminidase release inhibition rate of three independent experiments. Degranulation rates of maximum response when cells are treated with vehicle and DNP-BSA (DNP+) or minimum response when cells are treated with vehicle alone (DNP-) are shown in the figures. *p < 0.05, **p < 0.001, compared with β-hexosaminidase release inhibition rate of vehicle.

**Figure 6:**
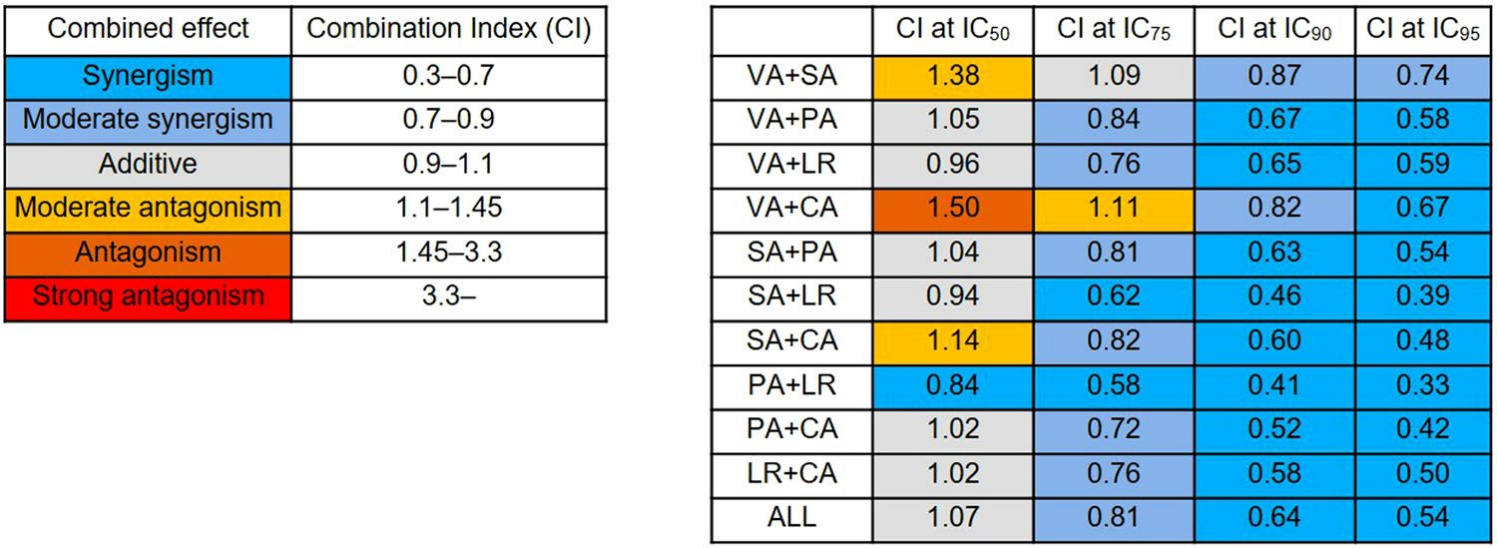
Combination index estimated from the combined effect of all active compounds on antigen-induced degranulation of RBL-2H3 cells. The effects of active compounds were tested in pairs (e.g., VA + SA). The two compounds were mixed at equal molar concentrations and the inhibitory effect of the mixture on β-hexosaminidase release was examined. The combination index (CI) at IC_50_, IC_75_, IC_90_ and IC_95_ was estimated from the β-hexosaminidase release inhibition rate. ALL: includes all five active compounds (VA + SA + PA + LR + CA). The combined effect was defined as follows: CI < 0.7, synergism; 0.7 ≤ CI ≤ 0.9, moderate synergism; 0.9 < CI < 1.1, additive effect; 1.1 ≤ CI ≤ 1.45, moderate antagonism; 1.45 < CI < 3.3, antagonism; 3.3 ≤ CI, strong antagonism. The evaluation criteria of the combined effect are listed and coloured.

We next examined the inhibitory effect of VA, SA, PA, LR and CA on antigen-stimulated degranulation (Fig. 4). All compounds inhibited IgE-mediated β-hexosaminidase release in a concentration-dependent manner. The half maximal inhibitory concentration (IC_50_) of these compounds on degranulation was calculated from the β-hexosaminidase release inhibition rate. The IC_50_ values of VA, SA, PA, LR, CA and TL were 0.88 mM, 1.63 mM, 0.60 mM, 1.48 mM, 0.43 mM and 0.44 mM, respectively. We further examined the inhibitory effect of these *ume* compounds on IgE-mediated degranulation of mouse bone marrow-derived mast cells (BMMCs) as normal mast cells. All compounds showed inhibitory effects on BMMC degranulation, similar to their effects on RBL-2H3 cells (Fig. 5). We further demonstrated that a mixture of the five active compounds exhibited an inhibitory effect on degranulation. Furthermore, the β-hexosaminidase release inhibition rate was determined by the combination of all compounds to evaluate the combined effect. The combination index (CI) was used to evaluate the combined effect at IC_50_, IC_75_, IC_90_ and IC_95_ (Fig. 6). Two-compound combination analysis showed most combinations had an additive effect at IC_50_. However, the CIs of all combinations were low at IC_90_–IC_95_.

**Figure 7:**
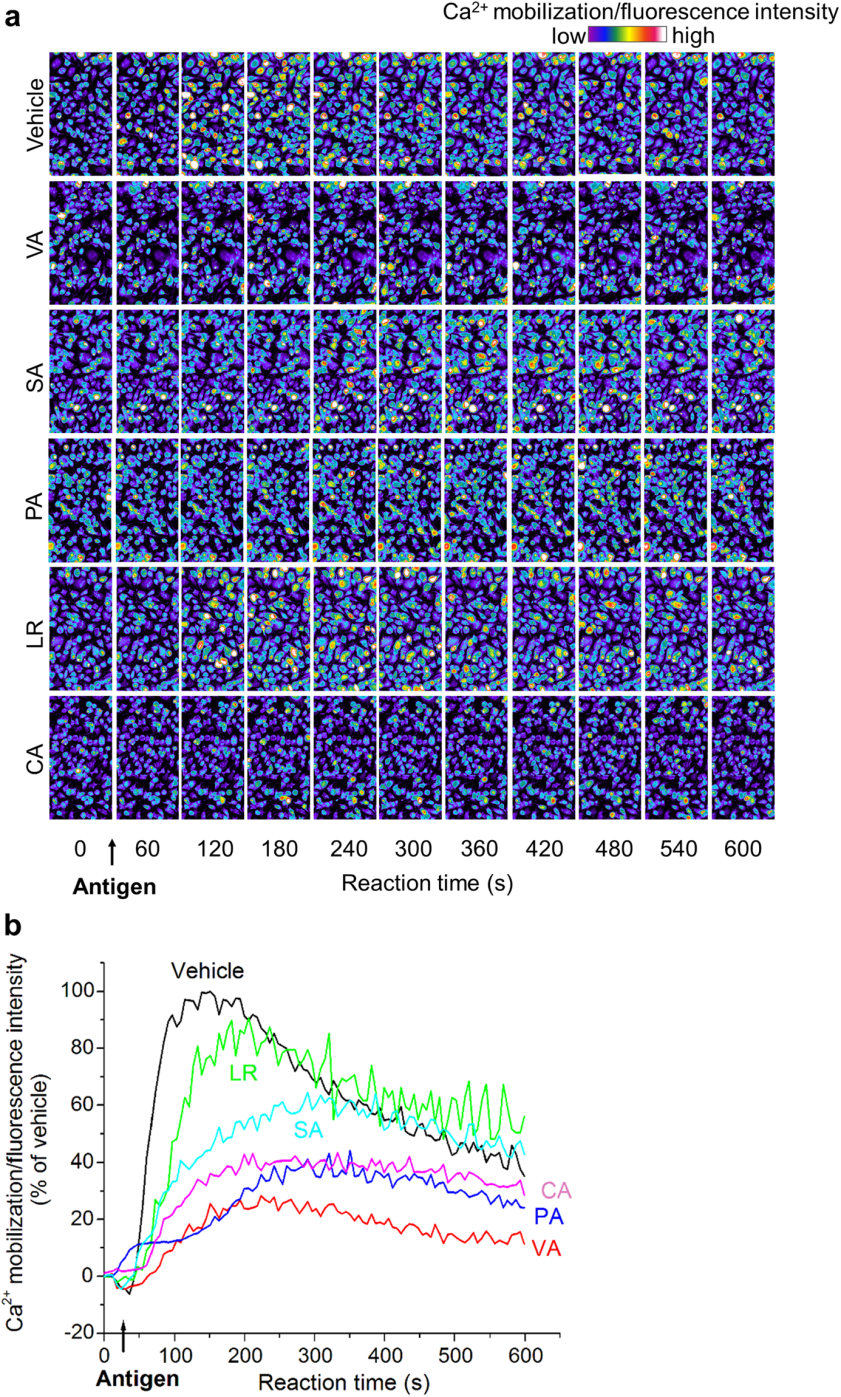
Effects of active compounds on intracellular Ca^2+^ mobilization of IgE-sensitized RBL-2H3 cells induced by antigen. IgE-sensitized RBL-2H3 cells were stimulated with DNP-BSA in the presence of active compounds derived from *ume* seed at 600 μM or vehicle (0.1% DMSO). (**a**) CLSM image shows the change in RBL-2H3 cells upon DNP-BSA stimulation. The crosswise direction indicates the reaction time of antigen stimulation. The bar represents fluorescence intensity from low (purple) to high (red). Scale bar: 20 µm. (**b**) Antigen-induced intracellular Ca^2+^ mobilization monitored by CLSM. The mean fluorescence intensities of the scan area were estimated from CLSM images. Arrow indicates antigen-stimulated time points. Results are representative of three independent experiments with similar results.

Upon antigen-stimulated degranulation, intercellular Ca^2+^ mobilization occurs due to the phosphorylation of proteins such as Lyn and Syk, which occurs following antigen-antibody cross-linking. Thus, to determine the mechanisms underlying the inhibitory effects of active *ume* compounds on antigen-stimulated degranulation, we examined the inhibitory effects of these compounds on intercellular Ca^2+^ mobilization (Fig. 7a). Cytosolic Ca^2+^ oscillation was stimulated by antigen challenge. Increased Ca^2+^ concentration is used as a universal signalling mechanism to control biological processes. Ca^2+^ signals are often presented to cells as Ca^2+^ oscillations, which encode information on both the amplitude and frequency of Ca^2+^ spikes^25^. To simplify the analysis, the fluorescence intensity of a Ca^2+^ indicator (fluo 3-AM) was measured in an area measuring 1024 × 1024 pixels monitored by confocal laser scanning microscopy (CLSM) (CLSM700; Carl Zeiss MicroImaging GmbH, Jena, Germany) (Fig. 7b). Intracellular Ca^2+^ mobilization occurred in non-treated antigen-stimulated cells, but was almost completely inhibited in VA-, PA- and CA-treated cells and partially attenuated in SA-treated cells. Conversely, intracellular Ca^2+^ mobilization was hardly inhibited by LR treatment.

**Figure 8:**
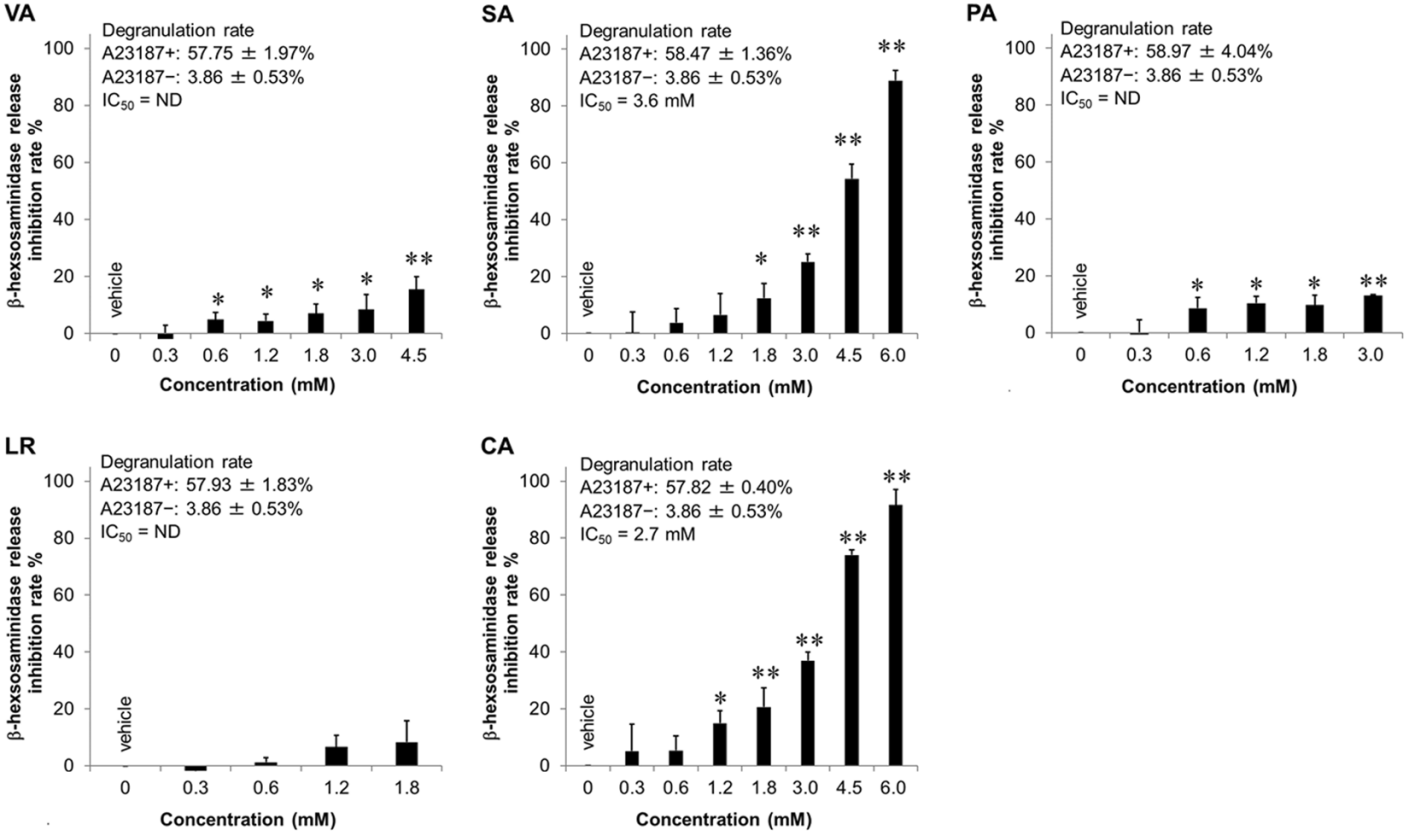
Inhibitory effects of active compounds derived from *ume* on non-IgE-mediated degranulation of RBL-2H3 cells. RBL-2H3 cells were stimulated with A23187 in the presence or absence of *ume* seed extracts. Data represent the mean ± SD (n = 3) of β-hexosaminidase release inhibition rate of three independent experiments. Degranulation rates of maximum response when cells are treated with vehicle and A23187 (A23187+) or minimum response when cells are treated with vehicle alone (A23187-) and IC_50_ are shown in the figures. ND: not determined. *p < 0.05, **p < 0.001, compared with vehicle.

We further examined the inhibitory effect of active *ume* compounds on a non-IgE-mediated stimulus, calcium ionophore A23187-induced degranulation (Fig. 8). Because Ca^2+^ ionophores, such as A23187, directly increase intracellular Ca^2+^ levels in cells, they can be used to study intracellular Ca^2+^-mediated events in mast cells^26^. The IC_50_ values of SA and CA were 3.6 mM and 2.7 mM, respectively. The IC_50_ values of VA, PA and LR were not determined. SA and CA inhibited non-IgE-mediated β-hexosaminidase release in a concentration-dependent manner. Similarly, VA and PA significantly inhibited non-IgE-mediated degranulation, but their effects were almost ineffective when compared with those of SA and CA. In contrast, LR hardly inhibited non-IgE-mediated β-hexosaminidase release.

**Figure 9:**
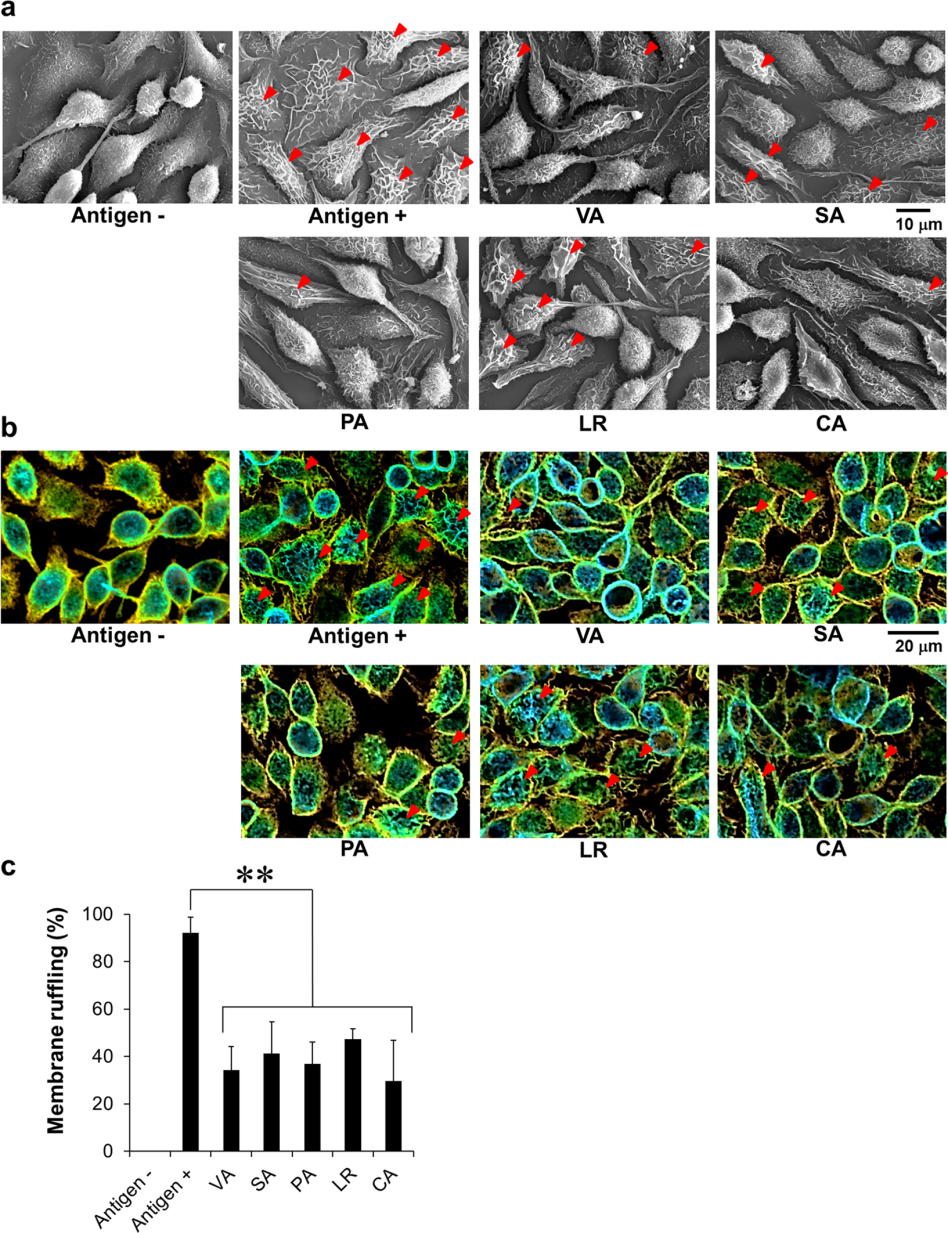
SEM and CLSM images of RBL-2H3 cells. IgE-sensitized RBL-2H3 cells were stimulated with DNP-BSA in the presence of active compounds derived from *ume* seed at 600 μM or vehicle (0.1% DMSO). (**a**) Cells were fixed, dehydrated and lyophilized. Cells were then coated with palladium and platinum and visualized by SEM. (**b**) Cells were fixed and stained with Acti-stain 488 phalloidin. Cells were visualized by CLSM. Red arrows indicate cells with membrane ruffling. (**c**) The percentage of cells with membrane ruffling was calculated from 5–7 SEM images. **p < 0.001, compared with antigen-stimulated vehicle.

A dramatic change in the actin cytoskeleton is induced by the activation of mast cells and basophils, which is indicated by membrane ruffling^27^. To examine the effects of active *ume* compounds on membrane ruffling, cell morphology was monitored by scanning electron microscopy (SEM) (Fig. 9a) and actin staining of RBL-2H3 cells was observed by CLSM (Fig. 9b). Membrane ruffling of IgE-sensitized RBL-2H3 cells was induced by DNP stimulation. All active *ume* compounds could inhibit membrane ruffling, similar to TL. In addition, significantly fewer cells with membrane ruffles were observed in cells treated with active *ume* compounds than untreated positive control cells (*p* < 0.001, Fig. 9c).

## Discussion

In the cross-sectional epidemiological pilot study, we investigated the association between *ume* intake and allergic symptoms in a specific area in Japan using a questionnaire survey. The proportion of women with allergic symptoms tended to be low in the high-intake group. Although it is the result of preliminary research, this result is consistent with the belief that *ume* is effective against allergic disease. Thus, to clarify the mechanism of *ume* on allergic reaction, we performed subsequent animal and *in vitro* studies.

We next demonstrated the effect of *ume* seed extract on PCA reaction in IgE-sensitized mice. Antigen challenge induced vascular permeability of mouse ears and mast cell degranulation was attenuated by oral administration of *ume* seed extract. These results indicated that *ume* seed extract inhibits mast cell-mediated allergic reactions.

We then identified the active *ume* compounds, which were fractionated by guiding as an inhibitory effect on IgE-mediated degranulation of RBL-2H3 cells. The five isolated active compounds were VA, SA, PA, LR and CA. A few functions of these compounds are already known. For example, VA has been shown to decrease fat accumulation by increasing fatty acid oxidation and decreasing adipogenesis in 3T3-L1 adipocytes^28^. VA is also known to inhibit mast cell degranulation^29^. However, the inhibitory mechanism of VA is not clearly understood. The present study showed that VA inhibits IgE-mediated degranulation, antigen-induced intracellular Ca^2+^ mobilization and membrane ruffling but not non-IgE-mediated degranulation. Therefore, VA affects a part of the event from antigen stimulation to intracellular Ca^2+^ mobilization.

Itoh *et al*. reported that LR derived from whisky congeners inhibits IgE-mediated degranulation of RBL-2H3 cells and reduces elevated intracellular Ca^2+^ concentrations^29^. In our study, LR hardly attenuated intracellular Ca^2+^ elevation in RBL-2H3 cells. A potential explanation for the discrepancy between the two studies may be the LR treatment period. In the previous study, the pretreatment period of LR was 30 min, whereas in our study it was 10 min. This result is consistent with the finding that LR had the lowest inhibitory effect of both β-hexosaminidase release and membrane ruffling among the five active compounds identified. In addition, LR did not inhibit non-IgE-mediated degranulation. Therefore, because LR does not inhibit non-IgE-mediated degranulation, it is expected to suppress the Ca^2+^-independent pathway, such as translocation of granules to the cell membrane^30^.

It has been reported that Ca^2+^ ionophore-induced mast cell degranulation is inhibited by CA but not SA^31^. However, the present study showed that both CA and SA inhibited IgE-/non-IgE-mediated mast cell degranulation. These compounds also inhibited antigen-stimulated Ca^2+^ mobilization. Therefore, CA and SA may suppress not only Ca^2+^ mobilization due to IgE-mediated stimulation but also events occurring after elevated Ca^2+^ concentration. To our knowledge, this is the first report to demonstrate an anti-allergic effect for SA.

PA has been demonstrated to have anti-inflammatory activities^32^, neuroprotective effects against oxidative stress^33^ and anti-cancer activities^34^. We also reported that PA has the potential to attenuate oxidative stress in human granulosa cells^23^. PA also inhibited both antigen-induced intracellular Ca^2+^ mobilization and membrane ruffling but not non-IgE-mediated degranulation, similar to VA. Therefore, PA likely affects a part of the event from antigen stimulation to intracellular Ca^2+^ mobilization. From the above findings, the five active *ume* compounds appeared to exhibit inhibitory effects through multiple mechanisms.

The IC_50_ calculated from the β-hexosaminidase release inhibition rate revealed the degree of degranulation inhibitory effect of these active compounds (in decreasing order): CA, PA, VA, LR and SA. Furthermore, the IC_50_ of CA (0.43 mM) was equivalent to that of TL (0.44 mM), an anti-allergic drug. Therefore, although the degree of inhibitory effect of these compounds on IgE-mediated degranulation is variable, synergistic effects of multiple compounds are expected as various compounds are actually contained in the edible part of *ume*. Thus, we next investigated the combined effect of the five active compounds. From the effect on β-hexosaminidase release and analysis of the combined effect, we found that the combination of the five active compounds maintained the ability to inhibit mast cell degranulation (Figs 4–6). We further evaluated the combined effect of active compounds by performing combination analysis. Two-compound combination analysis showed the CIs of all combinations were low at IC_90_–IC_95_. Thus, combinations of these active compounds should be used to acquire strong inhibitory effects on cell degranulation. From these results, it is expected that *ume*, including these compounds, may have the ability to attenuate type I allergic reactions. These findings are important for the future development of medicines and supplements for type I allergy.

In this study, the frequency of *ume* intake was associated with allergic symptoms in women. However, this study has limitations related to selection bias and its cross-sectional design. Additionally, we did not investigate dietary habits in detail. Since the survey area is a specific geographic area, the results cannot be directly generalized. Nevertheless, it has been reported that there is a sex difference in the prevalence rate of allergic diseases^35,36^. One reason for this difference is thought to be the influence of sex hormones, and female sex hormones such as oestrogen may be involved in susceptibility to anaphylaxis. For example, it has been reported that oestradiol enhances mast cell degranulation through extracellular Ca^2+^ influx by binding of oestrogen to mast cell membrane oestrogen receptor-alpha^37^. Additionally, it has been reported that the effect of oestradiol on anaphylaxis in mouse is related to enhanced vascular permeability, which is associated with oestradiol-mediated upregulation of endothelial nitric oxide synthase (eNOS) and nitric oxide (NO)^38^. The effect of *ume* and *ume*-related products suggested by the results of our study using human ovarian granulosa cell lines have been considered to be a regulation of sex hormone secretion^23^. From these views, *ume* intake may not only inhibit mast cell degranulation but also may influence the regulation of oestradiol or affect the regulation of oestradiol-mediated eNOS and NO, thereby exerting an anti-allergic effect in women. These views also may be related to the fact that the proportion of women with allergic symptoms in the high-intake group was lower than those in the middle- and low-intake groups, which was more prominent than in men.

In previous epidemiological studies, Enomoto *et al*. indicated ≥3 *ume* daily intake inhibits *H*. *pylori* infection, and they speculated the mechanism is related to the inhibitory effect of (+)-syringaresinol contained in *ume* on *H*. *pylori* motility^22,39^. Maekita *et al*. showed that ≥1 *ume* daily intake improves digestive motility symptoms^40^. The authors considered that increased alimentary fibres due to *ume* ingestion could also reduce digestive motility because *ume* fibres increase faecal output and improve gastrointestinal motility by promoting gastrointestinal tract emptying. Indeed, in experimental mouse studies, it has been reported that *ume* fibres possess faecal lipid excretion effects and faeces bulking effects, which affects the intestinal flora composition^41^. Recently, the correlation between allergic symptoms and intestinal microflora has been studied. Inhibition of an increase in bacterial species related to development of allergic symptoms may effectively prevent pollinosis^42^. Therefore, *ume* influences intestinal microflora and microorganisms, and daily *ume* intake may prevent allergy by maintaining the balance of intestinal microflora.

There remains the possibility that *ume* inhibits allergic reactions from degranulation to inflammation because we only examined the effect of *ume* on mast cell degranulation. Furthermore, *ume* may influence processes from antigen exposure to antibody production. At least five compounds derived from *ume* were found to inhibit IgE-mediated degranulation from studies using RBL-2H3 cells and BMMCs. The PCA reaction was also inhibited by *ume* extract. These findings suggested that *ume* intake suppresses mast cell degranulation. However, the concentration of active compounds contained in the *ume* extract administered in the animal experiment was about 10–100 times higher than that contained in one piece of *ume* ingested by humans. Because the animal experiment in this study only examined the presence or absence of an anti-allergic effect by single administration, the amount of *ume* active compounds does not necessarily need to be consistent with amounts ingested by humans. However, future studies should also investigate the inhibitory effect of low-dose and long-term administration of *ume* on the PCA reaction similar to the effect of *ume* on the regulation of oestradiol and oestradiol-mediated eNOS and NO. In addition, we have not studied the inhibitory effect of degranulation due to intake of *ume* active compounds in humans. Therefore, it is also necessary to conduct detailed clinical investigations including not only the association between *ume* intake and allergic symptoms but also the effect of *ume* on sex hormones and eNOS. It is important to clarify these points in future work.

## Conclusion

This cross-sectional epidemiological pilot study suggested that *ume* intake may be associated with reduced risk of allergic symptoms in women. One of the mechanisms involved in the anti-allergic effect of *ume* is inhibition of mast cell degranulation, which was revealed by the PCA test using IgE-sensitized mice as an *in vivo* model of type I allergy. In addition, we isolated active *ume* compounds that exhibited inhibitory effects on IgE-mediated degranulation by using RBL-2H3 cells and BMMCs, which were confirmed as VA, SA, PA, LR and CA. The *in vitro* study suggested that these compounds inhibited IgE-sensitized mast cell degranulation by attenuating intracellular Ca^2+^-dependent or -independent pathways. These results suggested that *ume* has the potential to inhibit mast cell degranulation and may be associated with reduced risk of allergic symptoms.

## Methods

### Cross-sectional epidemiological pilot study

This study was performed in accordance with the Declaration of Helsinki. The study protocol was approved by the Ethics Committee of Wakayama Medical University (Reference No. 1005). All participants provided written informed consent to participate in the study. The survey target was apparently healthy community-dwelling individuals living in the Kinan area, a well-known *ume*-growing region in Wakayama Prefecture. In December 2014, questionnaires were mailed to the 689 people who participated in Japan agricultural cooperatives-held community activities conducted in the target area in 2013. A stamped envelope was provided to encourage return of the questionnaire. We received the questionnaires from 586 participants (297 men, 289 women and 84% response rate) by March 2015. They were asked to answer the self-administered questionnaire including the following items about frequency of *ume* intake and allergy symptoms: ‘How often do you intake *umeboshi*?’ and ‘Do you have any allergies (symptoms including rhinitis during specific pollen seasons)?

Among our participants who typically consumed *ume*-related products, 95% of them ate pickled *ume* (‘*umeboshi*’ in Japanese). Other *ume*-containing products such as liquor, juice, jam and minor products were noted, however, the number of subjects who consumed these products and their intake frequency were low. In addition, these products were usually consumed with *umeboshi*. Thus, we judged that the intake frequency of other *ume* products was not necessary for analysis. Therefore, *ume* here means *umeboshi*.

In this study, after excluding participants with missing information (n = 23), the data of 563 participants (288 men and 275 women) were analysed. We divided 563 participants into 3 groups according to *ume* intake frequency: ≥1 *ume* daily (high-intake; 91 men and 92 women), <1 *ume* daily (middle-intake; 67 men and 67 women) and ≤2 *ume* weekly or none (low-intake; 130 men and 116 women). In this study, we conducted all analyses stratified by sex, as prior studies have indicated sex differences in some allergic reactions in human^43–46^. First, descriptive analyses were performed. Next, the odds ratios (ORs) and 95% confidence intervals (95% CI) of *ume* intake frequency on presence of allergy symptoms were calculated using multivariate logistic regression analyses adjusted for age (5 age categories: <40, 40–49, 50–59, 60–69 and ≥70 years), presence/absence of illness (except for allergic disease) and presence/absence of medication (including anti-allergic drugs). All statistical analyses were performed by using SPSS version 25.0 for Windows (IBM Corp., Armonk, NY, USA). *P* values < 0.05 were considered significant.

### Reagents

Mouse monoclonal anti-DNP antibody was purchased from Sigma-Aldrich (St. Louis, MO, USA). DNP-BSA was purchased from Biosearch Technologies (Petaluma, CA, USA). *p*-Nitrophenyl-N-acetyl-β-D-glucosaminide (*p*-NAG) was purchased from Merck Millipore (Darmstadt, Germany).

### Animals

Male ICR mice (4-week-old) and male BALB/c mice (7-week-old) were purchased from CLEA Japan (Shiga, Japan) and housed at 23 ± 1 °C and 55 ± 10% humidity under 12-h light/12-h dark cycles. Mice had access to laboratory rodent feed (Oriental Yeast, Tokyo, Japan) and water *ad libitum*. The experimental protocols of this study were approved by Wakayama Medical University Animal Care and Use Committee (Reference No. 679). All animal experiments were performed according to the Regulations for Animal Experiments at Wakayama Medical University.

### Passive cutaneous anaphylaxis reactions

ICR mice (5- to 6-week-old) were sensitized with 20 μL of anti-DNP-IgE/PBS (25 μg/mL) by intradermal injection in the ears with a 0.5-mL insulin syringe (Terumo, Tokyo, Japan). After 24 h, passive cutaneous anaphylaxis (PCA) reactions were induced by intravenous injection of 200 μL of DNP-BSA/PBS (0.5 mg/mL) containing 1% Evans blue. Samples or vehicle (water) were orally administered by feeding needle 1 h before antigen challenge. *Ume* extract was dissolved in water and TL was suspended in 0.5% methyl cellulose solution (Wako, Osaka, Japan). Mice were sacrificed under anaesthesia 30 min after antigen challenge, and their ears were removed.

For measurement of Evans blue extravasation, mouse ears were punched by an 8-mm biopsy punch (Kai Medical, Gifu, Japan) and punched ears containing extravasated dye were dissolved in 800 μL of 1 N KOH at 37 °C for 24 h. An equal amount of phosphoric acid/acetone (5:13, v/v) was mixed with ears dissolved in KOH and filtered by a 0.45-μm filter. The absorbance of the extract was measured at 620 nm. For histological observation, remaining ears were fixed in 10% formalin and stained with toluidine blue (paraffin-embedded tissue sections, 4-μm thick).

### Cell culture of rat mast cell line

The rat mast cell line RBL-2H3, which has been well-documented to release IgE-mediated histamine, established from peripheral blood with basophilic leukaemia^47^ was obtained from the Japanese Collection of Research Bioresources Cell Bank (Osaka, Japan). Cells were grown at 37 °C in 5% CO_2_ atmosphere in minimum essential medium (Life Technologies, Carlsbad, CA, USA), which was supplemented with 10% (v/v) fetal bovine serum (FBS; HyClone, Logan, UT, USA), 100 U/mL penicillin and 100 µg/mL streptomycin (Thermo Fisher Scientific, Waltham, MA, USA).

### Preparation of bone marrow-derived mast cells (BMMCs)

BMMCs were obtained by culturing bone marrow cells (BMCs) from 7-week-old male BALB/c mice (CLEA Japan). BMCs, derived from femur, were cultured at 37 °C in 5% CO_2_ atmosphere in Dulbecco’s modified Eagle medium (DMEM; Life Technologies) supplemented with 10% (v/v) FBS, 100 U/mL penicillin, 100 µg/mL streptomycin (Thermo Fisher Scientific), 5 ng/mL interleukin-3 (R&D Systems, Minneapolis, MN, USA), 5 ng/mL recombinant murine stem cell factor (PeproTech, Rocky Hill, NJ, USA) and non-essential amino acid (Life Technologies). After 4 weeks of culture, ≥95% of cells were confirmed to be mast cells, as assessed by flow cytometric analysis of cellular staining with anti-FcεRIα conjugated FITC antibodies (Miltenyi Biotec GmbH, Bergisch Gladbach, Germany) and anti-c-kit conjugated PE antibodies (Miltenyi Biotec GmbH) using FACSVerse (BD, Franklin Lakes, NJ, USA).

### β-hexosaminidase release assay of RBL-2H3 cells

For antigen-induced β-hexosaminidase release assay, RBL-2H3 cells were seeded at a density of 7 × 10^4^ cells/well in 96-well plates and cultured for 24 h. Then, cells were treated with 100 μL of anti-DNP IgE at a concentration of 100 ng/mL in culture medium for 18 h. Cells were then pretreated with 50 μL of samples or vehicle (0.1% dimethyl sulfoxide, DMSO; Sigma-Aldrich) in HEPES-buffered Tyrode’s solution (HT buffer; 140 mM NaCl, 2.7 mM KCl, 0.37 mM NaH_2_PO_4_, 12 mM NaHCO_3_, 0.49 mM MgCl_2_, 1.8 mM CaCl_2_, 25 mM HEPES and 5.6 mM D-glucose [pH 7.4]) for 10 min. Following pretreatment, cells were stimulated by addition of 50 µL of DNP-BSA (2 μg/mL) in HT buffer for 1 h at 37 °C.

For calcium ionophore-induced β-hexosaminidase release assay, RBL-2H3 cells were seeded at a density of 7 × 10^4^ cells in 96-well plates and cultured for 48 h. Cells were then pretreated with 50 μL of samples or vehicle (0.1% DMSO) in HT buffer for 10 min. Following pretreatment, cells were stimulated by addition of 50 µL of A23187 (10 μM) in HT buffer for 30 min at 37 °C.

To determine β-hexosaminidase release, supernatants were moved to new plates and remaining cells were lysed in 100 μL of HT buffer with 0.1% Triton X-100. Then, 25 μL of 3.3 mM *p*-NAG in 50 mM citrate buffer (pH 4.5) was added to each well and plates were incubated at 37 °C for 30 min. The reaction was stopped by adding 100 μL of carbonate buffer (pH 10.6). The absorbance due to *p*-nitrophenol from hydrolysis of *p*-NAG by β-hexosaminidase was measured at 405 nm by a microplate reader (Hitachi High-Technologies Corporation, Tokyo, Japan). The absorbance at 650 nm was measured as a reference wavelength. The β-hexosaminidase release inhibition rate was calculated as follows:

$$\begin{array}{rcl}{\rm{\beta }} \mbox{-} {\rm{hexosaminidase}}\,{\rm{release}}\,{\rm{rate}} & = & ({A}_{405}\,{\rm{of}}\,{\rm{supernatant}}-{A}_{650}\,{\rm{of}}\,{\rm{supernatant}})\\  &  & /\,\{({A}_{405}\,{\rm{of}}\,{\rm{supernatant}}-{A}_{650}\,{\rm{of}}\,{\rm{supernatant}})\\  &  & +\,({A}_{405}\,{\rm{of}}\,{\rm{cell}}\,{\rm{lysate}}-{A}_{650}\,{\rm{of}}\,{\rm{cell}}\,{\rm{lysate}})\}\end{array}$$

$$\begin{array}{rcl}{\rm{\beta }} \mbox{-} {\rm{hexosaminidase}}\,{\rm{release}}\,{\rm{inhibition}}\,{\rm{rate}}\,( \% ) & = & \{1-({\rm{\beta }} \mbox{-} {\rm{hexosaminidase}}\,{\rm{release}}\,{\rm{rate}}\\  &  & \times \,{\rm{of}}\,{\rm{sample}})/({\rm{\beta }} \mbox{-} {\rm{hexosaminidase}}\\  &  & \times \,{\rm{release}}\,{\rm{rate}}\,{\rm{of}}\,{\rm{positive}}\,{\rm{control}})\}\\  &  & /\,\{1-({\rm{\beta }} \mbox{-} {\rm{hexosaminidase}}\,{\rm{release}}\\  &  & \times \,{\rm{rate}}\,{\rm{of}}\,{\rm{negative}}\,{\rm{control}})\\  &  & /\,({\rm{\beta }} \mbox{-} {\rm{hexosaminidase}}\,{\rm{release}}\,{\rm{rate}}\\  &  & \times \,{\rm{of}}\,{\rm{positive}}\,{\rm{control}})\}\times 100\end{array}$$

Here, sample represents cells treated with samples, positive control represents cells treated with vehicle and DNP-BSA or A23187 and negative control represents cells treated with vehicle alone.

### β-hexosaminidase release assay of BMMCs

BMMCs were seeded at a density of 1.0 × 10^5^ cells/well in 96-well plates and cultured with anti-DNP IgE at a concentration of 100 ng/mL in medium for 18 h. Cells were then centrifuged at 1,000 rpm for 5 min and medium was removed. Cells were then pretreated with 50 μL of samples or vehicle (0.1% DMSO) in HT buffer. After 10 min, cells were stimulated by addition of 50 µL of DNP-BSA (2 μg/mL) in HT buffer for 1 h at 37 °C. For determination of β-hexosaminidase release, cells were centrifuged and supernatant and cells were separated. Subsequent operations and rate determination were carried out as described above for RBL-2H3 cells.

### Isolation and identification of active *ume* compounds

*Ume* seeds were a gift from Okahata Farm Co., Ltd. (Wakayama, Japan). The seeds were crushed, and kernels were removed. The kernel-removed seeds (described hereafter as ‘seeds’) were used for the experiment. The seeds (300 g) were extracted three times (for 3 h each) with methanol (900 mL). The methanol extract was filtered through a Whatman #1 filter paper and concentrated under reduced pressure.

The resultant methanol extract (46 g) was suspended in distilled water (1 L) and extracted with hexane (3 L), dichloromethane (3 L) and ethyl acetate (3 L) to obtain hexane, dichloromethane and ethyl acetate fractions. Each fraction was concentrated under reduced pressure to yield hexane (3049 mg), dichloromethane (670 mg) and ethyl acetate (1130 mg) fractions. Each fraction was dissolved in DMSO and used to measure the inhibitory effect on β-hexosaminidase release. Active fractions, based on inhibitory effects on β-hexosaminidase release, were further separated by column chromatography as described below.

The dichloromethane fraction (670 mg) was subjected to silica gel column chromatography and eluted with dichloromethane/acetone to separate six fractions (Fr. 1−6). Fr. 2 was further subjected to silica gel column chromatography and eluted with hexane/ethyl acetate to separate six subfractions (Fr. 7−12). Fr. 8 was further separated by preparative thin layer chromatography (TLC) with dichloromethane/acetone to yield compound 1 (0.6 mg). Fr. 4 was also further subjected to silica gel column chromatography and eluted with hexane/ethyl acetate to separate five subfractions (Fr. 13–17). Fr. 16 was separated by preparative TLC with dichloromethane/acetone to yield compound 2 (0.6 mg).

The ethyl acetate extract (129 mg) was subjected to silica gel column chromatography and eluted with a gradient of dichloromethane/MeOH to separate six fractions (Fr. 18−23). Fr. 20 was further subjected to C18 column chromatography (Nacalai Tesque, Kyoto, Japan) and eluted with a gradient of H_2_O/MeOH to separate eleven subfractions (Fr. 24–34). Fr. 27, 33 and 34 were subjected to preparative high-performance liquid chromatography (HPLC) to yield compounds 3 (1.5 mg), 4 (2.8 mg) and 5 (0.5 mg), respectively.

The chemical structures of compounds 1−5 were identified as vanillin, syringic acid, protocatechuic aldehyde, lyoniresinol and *p*-coumaric acid by high-resolution electrospray ionization mass spectrometry (HR-ESI-MS), proton nuclear magnetic resonance (^1^H-NMR) and direct comparison with an authentic sample. The HR-ESI-MS spectrum was obtained using a micrOTOF mass spectrometer (Bruker Daltonics, Billerica, MA, USA). The ^1^H-NMR spectrum was acquired using a Bruker AVANCE400 instrument (400 MHz; Bruker BioSpin, Billerica, MA, USA).

### Effect of *ume* compounds on cell viability

RBL-2H3 cells were seeded at a density of 7 × 10^4^ cells/well in 96-well plates and cultured for 48 h. Then, cells were treated with 100 µL of samples or vehicle (0.1% DMSO) in culture medium. After 70 min (equal to the period of β-hexosaminidase release assay), the medium was replaced with 100 µl of fresh medium, and 25 µl of 3-(4,5-dimethylthiazol-2-yl)-5-(3-carboxymethoxyphenyl)-2-(4-sulfophenyl)-2H-tetrazolium, inner salt (MTS; Promega, Madison, WI, USA) solution was added to each well. Cells were then incubated for 1 h at 37 °C in 5% CO_2_ atmosphere. The absorbance of each well was recorded at 490 nm using a microplate spectrometer because MTS is converted to a formazan product by dehydrogenase enzymes found in metabolically active cells. Cell viability was expressed as a percentage relative to the vehicle. Data are expressed as the mean ± SD of triplicate wells (n = 3).

### Synergistic effect analysis

The combined effects of active compounds were evaluated by using the combination index (CI) estimated from the median-effect theorem^48^. The CI and 50% inhibitory concentration (IC_50_) were calculated based on the β-hexosaminidase release inhibition rate using Compusyn software (ComboSyn, Inc., Paramus, NJ, USA). The combined effect was defined as follows: CI < 0.7, synergism; 0.7 ≤ CI ≤ 0.9, moderate synergism; 0.9 < CI < 1.1, additive effect; 1.1 ≤ CI ≤ 1.45, moderate antagonism; 1.45 < CI < 3.3, antagonism; and 3.3 ≤ CI, strong antagonism.

### Calcium imaging

Intracellular Ca^2+^ was monitored by CLSM using fluo-3 acetoxymethyl ester (fluo 3-AM; Dojindo, Kumamoto, Japan) as an indicator. RBL-2H3 cells (2.5 × 10^5^ cells/well) were subcultured on 8-well chamber slides (Iwaki, Tokyo, Japan) and sensitized with 100 ng/mL of anti-DNP IgE for 18 h. Cells were then incubated at 37 °C for 30 min with HT buffer in the presence of 3 μM fluo 3-AM, 0.04% cremophor (Wako) and 1.25 mM probenecid (Sigma). Before antigen challenge, cells were preincubated with 250 µL of HT buffer containing bioactive compounds from *ume* seed extract or DMSO for 10 min.

Ca^2+^ images were recorded using CLSM. Successive images were collected at 6-s intervals with a convert 20×/0.8 NA (numerical aperture) objective lens. Fluo 3-AM fluorescence was excited with a laser at 488 nm and an LP490 emission filter was chosen. After starting the time-lapse image scan, DNP-BSA was added to the well (final concentration of DNP-BSA = 1.0 μg/mL) at the indicated time point. Quantification of the fluorescence intensity of the scan area was performed using ZEN2009 software (Carl Zeiss).

### Observation of membrane ruffling

RBL-2H3 cells were seeded at a density of 7 × 10^4^ cells/well in 96-well cover glass bottom plates and cultured for 24 h. Then, cells were treated with 100 μL of anti-DNP IgE at a concentration of 100 ng/mL for 18 h. Cells were then pretreated with 50 μL of samples or vehicle (0.1% DMSO) in HT buffer for 10 min. After pretreatment, cells were stimulated by addition of 50 µL of DNP-BSA (2 μg/mL) in HT buffer for 1 h at 37 °C.

For CLSM imaging, cells were washed with PBS twice and fixed with 4% (w/v) paraformaldehyde for 10 min at room temperature. Following two washes with PBS, cells were permeabilized in 0.5% Triton X-100 in PBS for 5 min at room temperature. For actin cytoskeleton staining, cells were treated with Acti-stain 488 phalloidin (Wako) for 30 min. Cells were visualized by CLSM (LSM700, Carl Zeiss MicroImaging GmbH). Acti-stain 488 phalloidin was excited by a 488-nm wavelength argon-ion laser. Optical fluorescence signals of Acti-stain 488 phalloidin were observed using an emission filter (BP490-555, Carl Zeiss MicroImaging GmbH). Confocal images were acquired using Plan-Apochromat (20×/0.8 NA, Carl Zeiss MicroImaging GmbH) objective lens. On taking confocal images (optical tomography), Z-directional movement for optical sectioning of the entire specimen was controlled by the focus motor unit on axial scanning at 0.7–1.0 μm focus steps. CLSM image resolution was set at 1024 × 1024 pixels. The images taken using CLSM can be made into computer-assisted three-dimensional reconstructions using ZEN2009 software (version 6.0 SP2; Carl Zeiss MicroImaging GmbH). The three-dimensional images were converted to one-dimensional stacked images using ZEN2009 software.

For SEM, cells were prepared by a general method as previously described^23^. Briefly, cells were fixed with 2% glutaraldehyde in 0.2 M cacodylate buffer (pH 7.4) for 2 h at 4 °C. Cells were washed with PBS and dehydrated using a graded series of ethanol (50, 70, 90, 95 and 100%), 50% *tert*-butyl alcohol (in ethanol) and 100% *tert*-butyl alcohol. Then, dehydrated cells were lyophilized. Samples were coated with palladium and platinum using an E102 ion sputter coater (Hitachi, Tokyo, Japan), followed by SEM observation (Technex, Tokyo, Japan).

### Statistical analysis

Student’s *t*-test was used to analyse the results of animal and *in vitro* studies. *P* values < 0.05 were considered significant. All statistical analyses were performed by using JMP Pro 12 software (SAS Institute, Cary, NC, USA).
